# A Retrospective Analysis of Children and Adolescents With Diabetic Ketoacidosis in the Intensive Care Unıt: Is It Significant that the Blood Ketone Level Becomes Negative in Diabetic Ketoacidosis?

**DOI:** 10.7759/cureus.10844

**Published:** 2020-10-08

**Authors:** Murat Kangin, Mehmet Nur Talay, Sibel Tanriverdi Yilmaz, Edip Unal, Meliha Demiral, Muhammed Asena, Mehmet Nuri Ozbek

**Affiliations:** 1 Pediatric Intensive Care, Saglik Bilimleri University, Gazi Yasargil Training and Research Hospital, Diyarbakir, TUR; 2 Pediatric Endocrinology, Saglik Bilimleri University, Gazi Yasargil Training and Research Hospital, Diyarbakir, TUR; 3 Pediatrics, Saglik Bilimleri University, Gazi Yasargil Training and Research Hospital, Diyarbakir, TUR

**Keywords:** dibetes mellitus, diabetic ketoasidosis, ketone

## Abstract

Introduction: Diabetic ketoacidosis (DKA) is the most common cause of acute morbidity and mortality in children and adolescents with type 1 diabetes mellitus (T1DM). Because DKA management is associated with complications, endocrine communities have published guidelines and attempted to set standards for DKA diagnosis and management worldwide. In this study, for the patients followed up in the intensive care unit who have been treated according to DKA protocols, clinical and laboratory characteristics, differences between new and old diagnosed patients, and results of treatment were evaluated.

Methods: The records of 67 patients hospitalized in the pediatric intensive care unit for the past two years were reviewed retrospectively. Patients were grouped as newly diagnosed and old diagnosed diabetics.

Results: The mean age of the patients was 8.66 ± 5.0 years (3 months to 17.9 years) and 39 (58.2%) were male. Forty-five patients (67.1%) presented with mild DKA and 22 (33.9%) with severe DKA. Fourteen (63.6%) of the severe DKA cases were newly diagnosed with T1DM. Six patients had hyponatremia (corrected serum Na level <135 mmol/L) and five had hypernatremia (serum Na level >145 mmol/L). Only one of the hyponatremic patients had severe acidosis, while four of the hypernatremic patients had severe acidosis. At the 14th hour, blood glucose levels were below 200 mg/dl, blood ketones became negative in 5.8 hours, and at 9.1 hours, blood pH and/or HCO_3_ levels were normalized, recovery criteria were completed, and subcutaneous (SC) insulin injection was started. Of the patients, 38 (56.7) were newly diagnosed with T1DM. The mean age of newly diagnosed T1DM patients was smaller (7.40 ± 4.96) than those with old diagnosis, respiratory rates (RRs) were higher and pCO_2_ levels were lower on admission. Blood glucose, blood ketone negativity, acidosis, and Glasgow coma score (GCS) scores of the newly diagnosed T1DM patients improved later than the previous diagnoses. Only one patient under two years of age with a pH of 6.89 was given HCO_3_. None of the patients had symptomatic brain edema and death.

Conclusions: As a result, DKA is an acute and serious complication of diabetes, whose results are promising when managed only with minimal individual changes according to guidelines. Bicarbonate administration is not needed except in patients with very severe acidosis. Bedside blood ketone monitoring seems to be important because it allows for early enteral feeding.

## Introduction

Diabetic ketoacidosis (DKA) is the most common cause of acute morbidity and mortality in children and adolescents with type 1 diabetes mellitus (T1DM) [1-3]. The combination of hyperglycemia (>200 mg/dl), metabolic acidosis (pH < 7.3 and HCO_3_ < 15 mmol/L), and ketonemia (>3 mmol/L) is defined as DKA [2,3].

T1DM is a disease with an increasing incidence in children and adolescents worldwide [4-6]. In 16.5-78% of newly diagnosed T1DM patients, DKA is still the first cause of admission to the hospital [4-14]. Low socioeconomic conditions, lack of family history of diabetes, living in countries with a low prevalence of DM, and being young are risk factors for DKA in newly diagnosed patients with T1DM [3]. In Western societies, awareness training has significantly reduced DKA rates in newly diagnosed patients over the years [7,15]. However, even in countries such as Germany and Austria, newly diagnosed patients still present with DKA at rates of over 30%. In patients with a previously diagnosed T1DM, adolescent patients are at risk, and the most common cause is skipping the insulin dose, technical problems with the pump in patients using an insulin pump, and infection [3,16].

Factors constituting the clinical picture in DKA are, in addition to insulin deficiency, dehydration, and increased hormones such as adrenaline, cortisol, glucagon, and growth hormone that are effective against insulin. Patients also have serious intracellular dehydration due to intravascular hyperosmolarity and this dehydration develops over a long period [3,16]. Rapid reduction of intravascular hyperosmolality causes swelling of cells and hypokalemia, leading to an increase in morbidity and mortality rates associated with DKA [3,17]. Although mortality rates have declined from 0.15% to 0.3% over the years, there is still a mortality rate of 4-12% reported in some countries [3,14,18]. Despite the decline in mortality rates from DKA, it is still responsible for more than half of all deaths in children and adolescents with diabetes [19,20]. Asymptomatic brain edema in the majority of the cases has been demonstrated by imaging studies in small patient groups [21,22]. Nevertheless, symptomatic brain edema is seen in 1-5% of DKA cases, and it has been suggested that inappropriate fluid-electrolyte and insulin therapies may lead to brain edema, but controversy continues on this subject [22-24]. It has been suggested that great care is needed in intensive fluid replacement in the first hours of treatment [25].

As DKA management is associated with complications, endocrine and intensive care associations have published guidelines and attempted to set standards in DKA diagnosis and management. In this way, a significant decrease in morbidity and mortality rates due to DKA has been achieved [1-3].

In this study, an evaluation was made of clinical and laboratory characteristics, differences between new and old patients, and the results of treatment were evaluated. The DKA treatment protocol used in the intensive care unit was prepared by the Pediatric Endocrine and Diabetes Association in 2016 based on the International Society for Pediatric and Adolescent Diabetes (ISPAD) and European Society for Paediatric Endocrinology (ESPE) guidelines [2,3].

## Materials and methods

In this study, a retrospective analysis was made of the records of 67 patients hospitalized for DKA in Diyarbakır Gazi Yaşargil Training Hospital-Pediatric Intensive Care Unit between 2017 and 2018. Demographic characteristics, history, and physical examination findings, heart rate (HR, pulse), respiratory rate (RR), and blood pressure measurements were recorded in the patient's files. Fluid, electrolyte, and insulin treatments of the patients were arranged according to the ESPE and the ISPAD consensus report [2,3].

Newly diagnosed T1DM and previously diagnosed T1DM patients were classified according to their pH and bicarbonate levels. Those with pH < 7.1 and/or HCO_3_ < 5 mmol/L were classified as severe DKA, and a pH value between 7.1 and 7.3 and a bicarbonate value between 5 and 15 are defined as mild-moderate DKA. Newly diagnosed DKA has been used to describe patients who have not been diagnosed before, while the previously diagnosed DM is used to identify patients who were diagnosed before admission to intensive care and are currently receiving insulin therapy. When the patients were first admitted to the center or intensive care unit, they were separated according to whether they had normal saline loading or not and there was a difference between these groups.

Biochemistry parameters were taken at baseline and after 12 hours, and blood gas values measured at 0, 2, 4, 8, 12 hours and above were recorded. Blood gas measurements were taken from a capillary blood sample on a Siemens RAPIDPoint 500 (Siemens Healthcare Diagnostics, Inc., Newark, USA) device. Blood pH > 7.3 and/or HCO_3 _> 15 mmol/L was accepted as a recovery of acidosis and the time at which these values were reached was recorded as the time of acidosis recovery.

Blood ketone measurements were measured hourly at the bedside with the FreeStyle Optium Hβketone® instrument (Abbott Laboratories, Ltd., Berkshire, UK) in the capillary blood sample. Blood ketone level was recorded as negative (<0.6 mmol/L), trace level (0.6-1.5 mmol/L), or positive (>1.5 mmol/L). The time at which the blood ketone level <1.5 mmol/L was noted as the time of ketone recovery. Bloody glucose levels were measured in the capillary blood sample every hour with the OneTouch® device (LifeScan, Inc., Malvern, USA). The time when the blood glucose level fell below 200 mg/dl (11.1 mmol/L) was noted as the time of blood glucose recovery.

Statistical analysis

Data obtained in the study were analyzed statistically using SPSS 21.0 software (IBM Corp. Armonk, USA). In descriptive statistics, data were expressed as mean ± standard deviation (SD) values. The conformity of the data to normal distribution was assessed with the Shapiro-Wilk test. Comparisons between groups were made using the Mann-Whitney U test, and Student’s t-test. A value of p < 0.05 was considered statistically significant.

## Results

The evaluation was made of 67 DKA cases in this study. The clinical and laboratory findings of the patients at presentation are summarized in Tables 1 and 2. At the time of presentation, six patients had hyponatremia (corrected serum sodium level < 135 mmol/L) and five had hypernatremia (serum sodium level > 145 mmol/L). One of the hyponatremic patients and four of the hypernatremic patients had severe acidosis.

**Table 1:** Clinical and demographic data and time of healing parameters DKA: diabetic ketoacidosis, GCS: Glasgow coma score, HR: heart rate, SBP: systolic blood pressure, DBP: diastolic blood pressure, RR: respiratory rate.

	Established DKA (n:29)	Newly diagnosed DKA (N:38)	P-value	Mild-moderate acidosis	Severe acidosis	P-value	With normal saline loading	Without normal saline loading	P-value	Total (n:67)
Clinical and demographic data										
Age (month)	125.8±56.0	88.8±59.6	0.012	110.6±59.8	92.9±61.5	0.26	101.2±65.0	114.0±47.6	0.44	104.8±60.5
Gender (female/male)	9/20	19/19		18/27	10/12		20/28	8/11		28/39
GCS	11.6±1.2	11.7±1.5	0.82	12.3±1.1	10.3±0.8	0.00	11.7±1.4	11.5±1.5	0.66	11.7±1.4
HR(/min)	132.3±11.9	135.7±11.8	026	1309±11.8	141.0±8.8	0.001	134.7±12.5	133.2±10.3	0.64	134.2±11.8
SBP (mm/Hg)	103.6±7.0	100.0±8.1	0.06	10.3±7,5	100.0±8.2	0.249	100.9±8.0	103.1±7.1	0.31	101.5±7.8
DBP (mm/Hg)	58.5±6.4	56.1±6.3	0.134	57.8±6.3	55.7±6.5	0.219	56.9±6.6	57.6±5.8	0.68	57.1±6.4
RR (n/min)	27.3±4.0	31.15±6.45	0.007	28.2±5.2	320±6.1	0.01	30.1±6.3	278±3.8	0.16	29.4±5.8
Recovery time of healing parameters (hour)										
Glucose (mmol/L)	11.86±6.97	15.87±8.71	0.047	10.8±4.5	20.8±9.8	0.00	14.2±8.8	13.7±6.3	0.83	14.1±8.1
Blood ketone (mmol/L)	4.90±3.53	6.58±4.64	0.10	4.1±2.5	9.2±4.9	0.00	5.5±4.4	6.6±3.7	0.34	5.8±4.2
Acidosis (HCO_3_) (mmol/L)	7.24±5.69	10.42±7.57	006	5.9±3.3	153±8.1	0.00	9.0±7.7	9.1±4.7	093	9.1±7.2

**Table 2:** Laboratory findings DKA: diabetic ketoacidosis.

	Established DKA (n:29)	Newly diagnosed DKA (N:38)	P-value	Mild-moderate acidosis	Severe acidosis	P-value	With normal saline loading	Without normal saline loading	P-value	Total (n:67)
Laboratory data										
Glucose (mmol/L)	22.92±9.90	24.11±7.13	0.57	22.9±8.9	24.9±7.1	035	23.2±8.3	24.4±8.8	0.59	23.5±8.3
HbA1c (%)	10.9±3.0	10.9±2.9	0.66	10.9±2.8	10.8±2.1	0.81	10.6±2.8	11.5±2.1	0.22	10.9±2.6
Urea (mg/dl)	27.74±15.74	27.03±14.57	0.85	25.4±11.1	30.9±20.3	0.163	28.1±16.4	25.1±10.2	0.46	40.8±7.8
Creatinine (mg/dl)	1.01±0.91	3.02±13.16	0.45	0.9±0.5	0.6±0.3	0.146	0.8±0.2	0.9±0.3	0.17	0.86±0.3
Sodium (mmol/L)	135.06±5.55	135.50±6.08	0.76	134.4±5.5	13±6.1	0.097	134.8±5.4	136.4±6.7	0.31	135.3±5.8
Potassium (mmol/L)	4.35±0.72	4.19±0.83	0.39	4.3±0.6	4.1±1.0	0.403	4.3±0.8	4.0±0.6	0.17	4.23±0.7
Chloride (mg/dl)	104.98±7.16	106.61±7.29	0.37	103.7±6.6	110.5±6.3	0.00	106.0±7.6	105.7±6.0	0.87	105.9±7.2
Phosphorus (mg/dl)	3.20±1.02	3.44±1.27	0.54	3.6±0.8	2.8±1.4	0.014	3.4±1.3	3.2±0.9	0.51	3.29±1.2
Phosphorus at 12 hours (mg/dl)	3.46±0.97	3.71±1.92	0.74	3.8±1.4	3.1±1.9	0.313	3.6±1.7	3.2±0.8	0.69	
pH	7.13±0.12	7.13±0.17	0.98	7.2±0.0	6.9±0.0	0.00	7.1±0.1	7.1±0.1	0.87	7.13±0.14
pCO_2_	24.44±5.81	20.26±5.03	0.002	22.8±5.2	20.4±6.4	0.11	22.2±5.9	21.5±5.1	0.67	22.0±5.7
HCO_3_ (mmol/L)	8.42±2.65	7.51±3.70	0.26	9.4±2.5	6.1±7.7	0.01	7.9±3.4	9.4±7.8	0.28	8.3±5.0
Ketone (mmol/L)	5.28±1.55	5.59±1.38	0.44	4.4±2.5	3.2±2.8	0.065	4.4±2.4	3.08±3.1	0.05	4.07±2.6

Of the total patients, 45 (67.1%) presented with mild-moderate DKA and 22 (33.9%) with severe DKA. Of these 22 patients, 14 were newly diagnosed T1DM. Severe DKA patients had a worse GCS, and higher HR and RR. Blood pH, HCO_3_, pCO_2_, and serum phosphorus levels were lower in patients with severe DKA than in those with mild to moderate DKA, and blood ketone, serum sodium, chlorine, urea, and creatinine levels were higher. Improvements took longer in cases with severe DKA (Tables 1 and 2).

Newly diagnosed T1DM was determined in 38 (56.7%) patients and 29 (43.3%) had been diagnosed previously. The newly diagnosed T1DM patients were younger at a mean age of 7.40 ± 4.96 years and had higher RRs and lower pCO_2_ levels at presentation (Table 1). The blood glucose, blood ketone level, acidosis, and GCS scores of the newly diagnosed T1DM patients improved later than those with a previous diagnosis (Table 2).

**Figure 1:**
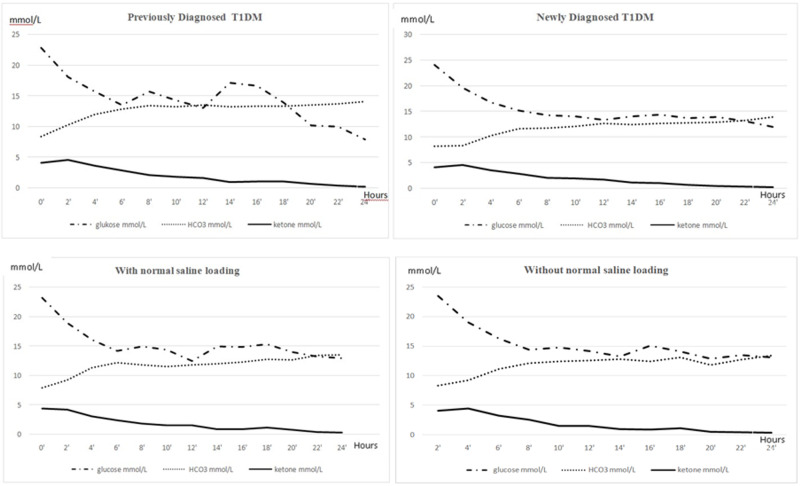
Hourly levels of blood glucose (mmol/L), blood ketone (mmol/L), and HCO3 (mmol/L) between groups

The earliest normalized parameter in patients was blood ketone. The mean blood glucose level of the patients was <200 mg/dl in 14.1 ± 8.1 hours of admission to ICU. Blood ketones became negative at 5.8 ± 4.2 hours, and blood pH and/or HCO_3_ levels were normalized at 9.1 ± 7.2 hours (Figure 1). When the recovery criteria were met, a subcutaneous (SC) insulin injection was initiated.

Hypoglycemia developed in only three of the patients who were followed up, however, 35 (52%) patients' dextrose rates were increased because their blood glucose decreased at a rate above 75 mg/dl per hour. Eleven patients developed hypochalemia and the amount of potassium in the infusion fluid was increased to 60 mmol/L. 

Bicarbonate was administered to one patient aged less than two years, with a pH of 6.89. Symptomatic brain edema and death were not observed in any patient.

## Discussion

In this study, evaluations were made of 67 DKA cases that were followed up in our intensive care unit in the last two years, and of these, 38 were newly diagnosed with T1DM. In Turkey, especially in our region, the majority of newly diagnosed patients are admitted with the clinical presentation of DKA [9,10]. In Western societies, especially in northern countries, there has been a significant reduction in the frequency of DKA in newly diagnosed T1DM [12,15]. In the Environmental Determinants of Diabetes in the Young (TEDDY) study, approximately 8000 children with human leukocyte antigen (HLA) risk genotype at birth were followed up from the age of three months, and the frequency of DKA was reported as 13.1% [15]. However, the frequency of DKA in newly diagnosed patients in countries such as Canada and Poland is reported to be over 20%, and in countries such as Germany and Austria more than 30% [4,5].

In a study conducted in Israel, the frequency of DKA in newly diagnosed patients was reported as 42%, but this rate was reported to be 21% in secular communities and 62% in highly religious communities [26]. It is clear that studies to increase awareness and close monitoring of the population at risk will provide a significant reduction in the frequency of DKA at the time of diagnosis. In children and adolescents with diagnosed T1DM, the incidence of DKA in western populations is between 5% and 7.1% [7]. In the current study, DKA was observed mostly in the adolescent age group in the previously diagnosed patients, but it was observed at younger ages, as expected, in newly diagnosed patients (Table 1). DKA was more severe in our newly diagnosed patients and improved later. Similarly, in a study reported from Canada, more severe DKA was observed in newly diagnosed patients [27]. This may be related to prolonged exposure to insulin deficiency as a result of delayed diagnosis [28].

Of the 22 patients with severe acidosis, 14 (63.6%) were newly diagnosed. The rates of severe acidosis in newly diagnosed patients have been reported to range from 5.9% to 42% [4,6,15,26,27]. The more frequent occurrence of severe acidosis in newly diagnosed patients is associated with a delay in diagnosis [28]. Hypernatremia was more common in patients with severe acidosis than hyponatremia. Hyponatremia is a factor facilitating the development of brain edema [3]. The presence of hypernatremia in patients with severe acidosis in the current cohort was considered to have decreased the risk of symptomatic brain edema because symptomatic brain edema did not develop in any of the patients with severe acidosis and no sequelae were observed in the early period.

The most important and controversial area of DKA management is fluid and electrolyte therapy [3,23,24,29,30]. According to the degree of acidosis, the fluid, which was calculated to have 5-10% deficiency in addition to the maintenance, was given to the patients in the current study at an equal rate in 48 hours. Insulin infusion of 0.05-0.1 unit/kg/hour was started one hour after fluid replacement. With this treatment protocol, blood ketone level decreased below 1.5 mmol/L in a mean of 5.8 hours, and the symptoms of ketonemia, nausea, and abdominal pain improved. In this way, patients were able to receive oral fluids, and most patients did not need intravenous fluids after 12-16 hours. As urine ketone reflects possible high blood ketone before diagnosis in DKA, it cannot be detected when blood ketone improves [3,29]. Normalization of blood ketone leads to earlier oral fluid intake and enables an earlier transition to SC insulin. As it was possible to follow up blood ketone in the current study patients, oral intake decisions could be made earlier and the transition to SC insulin was made at 11 hours on average. With the exception of two cases, the patients were discharged from the intensive care unit within the first 24 hours. The improvement in blood glucose levels as late as 14 hours was due to the relatively high level of dextrose in the given fluid to prevent hypoglycemia because normalization of blood glucose before acidosis improves will require a reduction in the insulin dose given and delay the recovery of acidosis. Nevertheless, the acidosis of the current patients improved in nine hours before the blood glucose level improved. pH > 7.3, HCO_3 _> 15 mmol/L, and blood ketone levels returning to normal are defined as healing criteria in DKA. The observation of ketonemia as the earliest resolving parameter in our cohort indicates that effective fluid and insulin therapy was administered. If the ketone level is accepted as a criterion for recovery and the patients begin to be fed early, we think that early discharge of the patients from the intensive care unit may be possible by switching to SC insulin treatment at an earlier stage. It is thought that the patient will have the chance to be discharged from the intensive care unit earlier after the prospective studies to be carried out on this subject, by opening the oral feeding way of the patient, without waiting for a full recovery in the pH value with the normalization of the ketone level.

In a prospective Pediatric Emergency Care Applied Research Network (PECARN) FLUID study evaluating the effect of different fluid regimens in DKA, it was found that no rapid or slow administration of 0.45% and 0.9% sodium chloride led to any neurological disorder [24]. In recent years, it has been believed that there is no relationship between fluid replacement therapy in DKA and the development of brain edema. Vasogenic and cytotoxic causes are more prominent in the etiopathogenesis of brain edema [23,29]. In the current study, the absence of significant hyponatremia and symptomatic brain edema as a result of slow administration of fluid at a concentration of 0.45% supports this thesis.

Another controversial area in DKA is initial fluid therapy [2,3,23,30]. There has been reported to be no difference between the administration of 10 ml/kg and 20 ml/kg at one hour [23]. It has even been suggested that loading fluid is only required in case of shock at the beginning, and if the patient does not have shock findings, loading fluid is not needed [17,30]. In the current study, no difference was observed between the results of patients with and without fluid loading (Table 2). It can be considered that replacement should be initiated with a bolus of 10 ml/kg/hour as recommended in consensus reports. However, it does not seem to need to be repeated at higher infusion rates except for shock patients [23,24].

After urine output was observed, 40 mmol/L potassium was added to the given fluid. To avoid hypophosphatemia and hyperchloremia, half of the potassium replacement was prepared as KCL and the other half as KPO_4_. However, in patients with severe acidosis and no normal saline loading at baseline, the mean serum phosphorus levels in the biochemistry at 12 hours were <3.5 mg/dl. The observed hypophosphatemia did not cause any symptoms except fatigue exceeding 48 hours in two patients with severe acidosis whose phosphorus level was <2.5 mg/dl.

## Conclusions

DKA is an acute and serious complication of diabetes, but the results are promising when it is managed in accordance with guidelines, only making minor individual changes. Bicarbonate is not needed except for patients with very severe acidosis. When it is seen that the ketone level returns to normal before other parameters are recovered as a result of the blood ketone measurements made at the bedside, the blood ketone measurement seems important because the oral fluid intake and nutrition of the patient can be started earlier.
